# Finding the Victim of Abuse: A New Frontier of Physicians' Liability? Data From a Local Italian Experience on Minor Maltreatment

**DOI:** 10.3389/fped.2020.00309

**Published:** 2020-06-19

**Authors:** Emanuela Turillazzi, Chiara Toni, Sara Turco, Marco Di Paolo

**Affiliations:** Section of Legal Medicine, Department of Surgical, Medical, Molecular Pathology and Critical Area, University of Pisa, Pisa, Italy

**Keywords:** minor maltreatment, minor abuse, mandatory reporting, omitted diagnosis, professional liability

## Abstract

Violence toward minors is a widespread phenomenon and effective programs are desperately needed to prevent it. Data from the literature showed that underreporting child/adolescents abuse has become a widespread phenomenon, exposing minors to additional harm from further potentially dangerous situations. It is proved that systematic screening and standardized procedures for minors presenting at emergency departments with the suspicion of abuse might increase the detection rate, reducing the risk of underreporting. In Italy a system of mandatory reporting is in place, and it is considered to be crucial in detecting abuse and preventing further harm to children. In this paper we report our experience with a regional (Tuscany) project named “Codice Rosa” (Pink code) introduced in 2014 with the aim to treat and protect the most vulnerable bracket of the population. We present data concerning the access of minors for suspected abuse at the emergency room of the local hospital, focusing on a case of omitted diagnosis leading to further violence episodes. According to our experience, since the introduction of the “Pink Code” there have been 43 cases of reported child abuse, with an increasing trend throughout these years (from 1 reported event in 2015 to 16 reported events in 2018). Despite the limited number of our population, the increasing trend in the reported events was particularly evident for bullying cases (*n* = 0 in 2015; *n* = 4 in 2018). Despite data are still limited, the procedure proved effective in preventing child abuse, though it could still be implemented. Minor abuse and maltreatment are important health issue globally which can lead to significant physical and psychological morbidity. Implementing knowledge of healthcare professionals on how to deal with child abuse and introducing educational programs on recognition, treatment and report of child abuse is mandatory not only to prevent missing diagnosis of child and minor maltreatment, but also to reduce the risk of professional liability on different bases.

## Introduction

Violence against children and adolescents is a widespread phenomenon. Data on the prevalence of violence are different by country, depending on the available documentation systems (1). In 2017 the WHO reported that up to 1 billion minors between 2 and 17 years of age have suffered some forms of violence (physical, emotional, or sexual) (2); in 2013, the same organization estimated that in Europe, 44 million (about 22.9%) have been victims of physical violence, while 55 million (29.6%) have been victims of psychological violence (3). Data from Italy indicates that violence affecting minors is a widespread phenomenon and that different typology of violence exist: sexual and physical violence, violence in the home, in the school and in the community (including on—line) (4).

Violence and abuse prevention comprises different international and national programs and strategies, aiming to reduce violence against children and adolescents. Complete understanding and knowledge of the phenomenon are essential. Concerns for the plight of the maltreated minors spread across national and international levels to a very high attention both in medical, legal and social environments. In this context, to give impetus and sanction that suspected maltreatments be reported to authorities, many state legislatures passed minor maltreatment and abuse reporting statutes. However, data from the literature showed that underreporting child/adolescents abuse has become an epidemic (5, 6). Due to this alarming phenomenon, minors would remain in potentially dangerous situations subject to additional harm. In fact, abuse often consists of a series of incidents rather than a single event, and unfortunately many cases are reported after several medical evaluations (7, 8).

As many other European countries, Italy has included in the criminal codes the general obligation for health care professionals to report knowledge (suspicion, or presumption) of a crime, also imposing penalties when failing to do so. A system of mandatory reporting is provided and considered to be of pivotal significance in detecting abuse and preventing further harm to children. The Italian law requires health care providers (who are considered “mandatory reporters”) to make a report to the Public Prosecutor's offices when they have a reasonable suspicion that a minor is abused or neglected or has suffered some form of violence (both physical and psychic). The report will trigger an investigation on the case aimed both at punishing the possible culprit and at preventing the minor from continuing to be abused. All health professionals are called to this duty, covering both those who are routinely in contact with children and adolescents, and those who may only occasionally come into contact with them in their work.

In the Italian region Tuscany, in 2012 a project named “Codice Rosa” (Pink Code) was set up, aimed to give protection to the most vulnerable people, including minors, more likely candidates to become victims of abuse or maltreatment. The project's main purpose was to standardize the public responses in the event of abuse and maltreatment, providing immediate operative responses when the victim seeks help at the emergency department, guaranteeing assistance and protection for the victim and facilitating the connection among the different network subjects in the later steps (9).

Following positive achievements, the regional Committee has extended the project units through Resolution n. 339/2013, achieving its complete diffusion in 2014. Our local hospital established a special procedure for minors in 2016, operating across the hospital's activities to guarantee effective treatment as well as a diagnostic classification of the individual child who is victim of abuse. At the same time, it carried out preventive action and early diagnosis of warnings connected to abuse. Finally, the procedure promotes clinical and legal processes cooperating with Territorial Services and competent institutions. It is formed of different professional figures—pediatricians, gynecological pediatricians, psychologists, psychotherapists, surgeons, nurses, social workers—who collaborate to select the best form of treatment for each individual case. Taking charge of a single case also entails undertaking the responsibility to inform the Judicial Authority about possible felonies and child protection.

In this paper we report the experience from 2015 to 2018 concerning the access of minors for suspected abuse at the emergency room of the local hospital, focusing on a case of omitted diagnosis of bullying leading to further violence episodes.

## Materials and Methods

We retrospectively analyzed 4 years' medical charts relating to all accesses due to suspected minor maltreatment at the Emergency Unit of the local hospital. The data were anonymised at an early stage of the study process.

## Results

**Figure 1:**
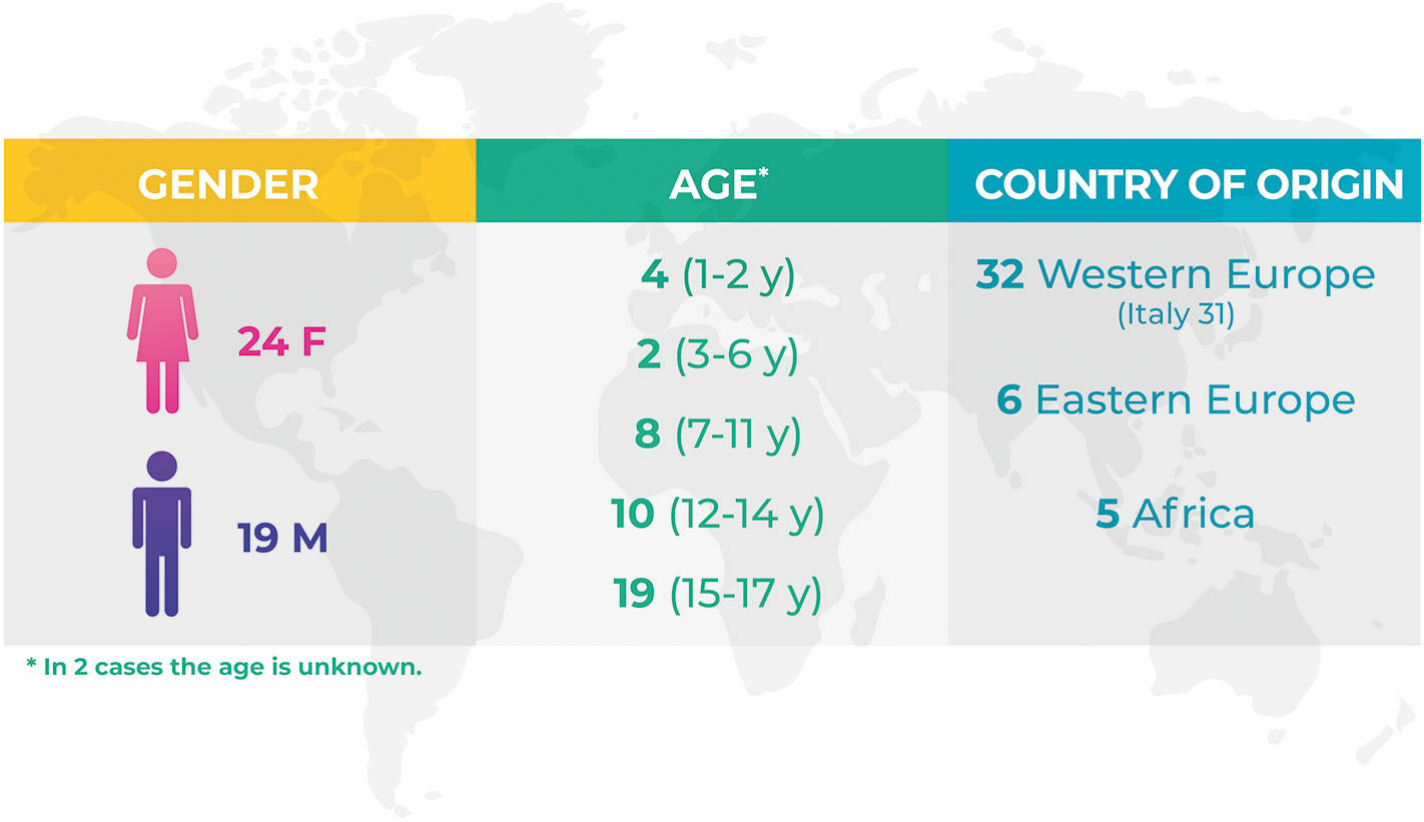
Demographics data of the reported Pink Code events.

**Figure 2:**
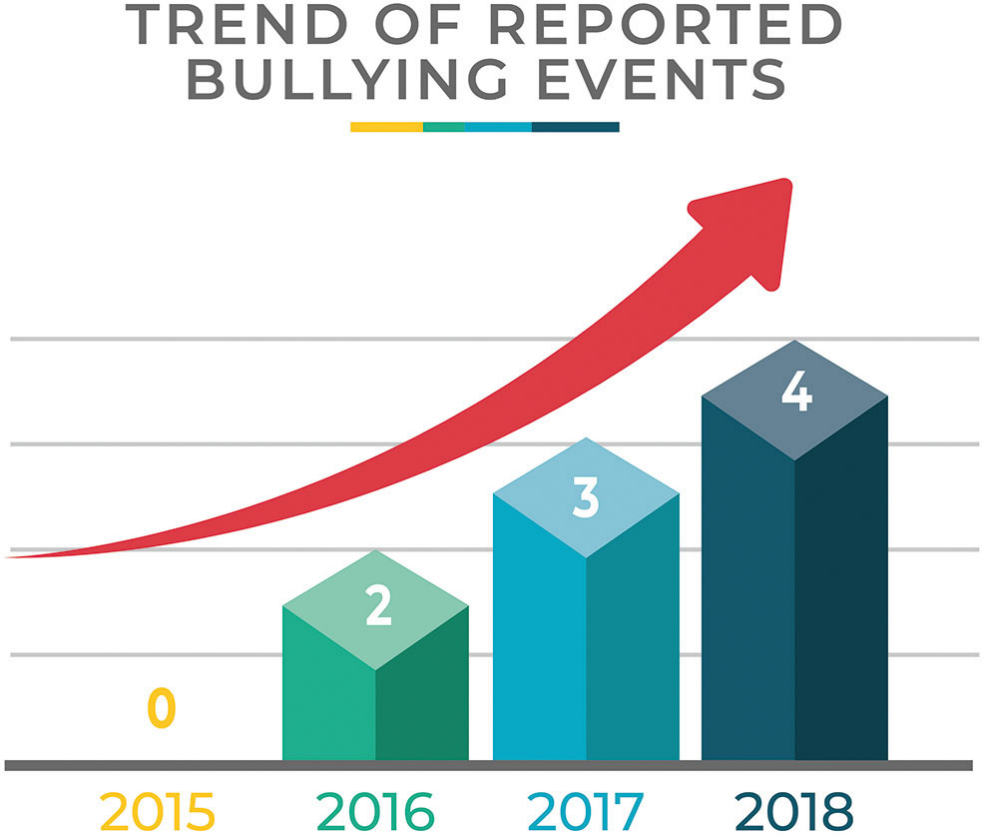
Number of reported bullying events per year.

Demographics data are shown in Figure 1. Table 1 shows the number of events/year and the type of event compared to the total amount of minors' accesses to the Emergency Room. Figure 2 focuses on the cases of bullying showing the number of events/year.

**Table 1:** Number of events/year and the type of event compared to the total amount of minors' accesses to the Emergency Room.

**Year**	**Type of event**					**Number of reported events**	**Minor's access to ER**
	**Violence**	**Abuse**	**Bullying**	**Violence and abuse**	**Neglect**		
2015				1		1	15.292
2016	7	3	2	1		13	15.479
2017	6	3	3		1	13	15.340
2018	8	4	4			16	15.293

In our casuistry, in a 4-year-period, from 2015 to 2018, 43 minors presented with the suspicion of violence and maltreatment. Data show that, starting from the activation of the standardized procedure, a significant increase in the number of events reported occurred: only 1 in 2015, when the procedure was recently approved, 13 in 2016 and 2017, 16 in 2018. The main reported event was violence (*n* = 21), which included beating by family members or by other adults outside the family, followed by cases of sexual abuse including sexual harassment (*n* = 10), bullying (*n* = 9), violence and abuse (*n* = 2) which included a history of beating by family members or other adults and sexual abuse, neglect (*n* = 1) represented by the case of an orphan without guardians.

The increasing reporting rate since the introduction of the “Pink Code” procedure was particularly evident for bullying, as in 2015 no case was reported, while in 2016 two cases were documented, growing to three cases in 2017, and four cases in 2018. In the first semester of 2019 (data not shown in Table 1 as incomplete), 7 cases of bullying were reported.

Among these cases, one deserves special attention as it demonstrates the importance of prompt recognition of the maltreatment in order to avoid the repetition of the same to the detriment of the minor. The case concerns an adolescent who accessed to the emergency room following a blunt, accidental trauma to the left hand, occurred while the adolescent was playing with a school friend. Stiffness of the proximal interphalangeal joint and generalized swelling of the ring finger was present; radiographic investigations demonstrated a small fracture at the end of the metacarpal. Non-operative management was instituted. The “Pink Code” procedure was not activated and the case was not reported to the competent authority. Some years later, the adolescent presented to an emergency department following a blunt lower abdomen trauma at school. Despite the correct treatment, the damaged organs did not make a full recovery, thus resulting in a permanent damage. Unfortunately, also in this case the pink code was not activated.

Some time later, the teenager told their mother that they had been bullied for many years at school. This initially took the form of verbal taunts from other children. The teenager described being surrounded and insulted on several occasions by groups of other adolescents and continued to have nightmares about these episodes. The situation deteriorated and the teenager became the target of physical bullying.

The physical violence culminated in two more serious episodes that led to hospitalization: the blunt trauma to the hand and to the lower abdomen. Fears around not being believed, and concerns about being called a “snitch” having to continue to face those who perpetrated the offense were the motivations that had led the boy to keep silence on the facts.

## Discussion

This study highlights a cascade of negative events that occurred to a young boy who had been bullied for several years at school, suffering significant injuries leading to hospital access. Following the first undiagnosed episode, the boy was left in the school violent environment, leading, some years later, to a severe episode (testicular blunt trauma) with permanent physical harm. Significance of minor abuse detection and reporting is stressed.

Minor abuse and maltreatment are important health issue globally which can lead to significant physical and psychological morbidity (10, 11). An alarming part about this issue is that a great proportion of events are undiagnosed and unreported. In our case too, maltreatment had reoccurred since it was left unreported in the beginning.

The reasons that professionals may fail to report child maltreatment fall into different categories (12, 13), among which failure to recognize maltreatment is assumed to be very significant. Child and adolescents' maltreatment seems underreported by professionals, mainly due to non-recognition (14) and the lack of confidence that reporting would improve patient outcomes (15). In their report from a survey of pediatricians attitudes in identification and reporting of child abuse, Flaherty et al. (6) showed a high variability in physician's response to ambiguous clinical scenarios, reflecting the difficulty physicians have in determining whether an injury was caused by abuse. Studies evaluating agreement regarding diagnosis of abuse between experts and non-experts reported significant differences between the two groups (16, 17). Other studies confirmed that uncertainty about the “diagnosis” is a key factor in preventing physicians from reporting (18, 19). An Italian study highlighted a scarce knowledge on the behalf of pediatricians and general practitioners regarding how to deal with child abuse (20).

Lack of educational programs on recognition, treatment and report of child abuse is recognized as pivotal in missing diagnosis of child and minor maltreatment (11). In fact, it is well-known that systematic screening and standardized procedures for minors presenting at emergency departments with the suspicion of abuse might increase the detection rate (21, 22). Furthermore, there is an enormous variability in determining whether an individual suspects abuse, the level of suspicion, and whether he or she believes this level of suspicion should trigger a report. Finally, a great variability in how people define the threshold for mandated reporting of suspected child abuse still exists (23, 24). Overall, the majority of studies included in a meta-synthesis by McTavish et al. found that mandatory reporters had negative experiences with the reporting process and that research on the effectiveness of this process is urgently needed (25).

Identifying suspected abuse and reporting reasonable suspicions to the competent authorities can be one of the most challenging and difficult tasks for the health care professionals. When failure to identify and report a suspected episode of maltreatment results in further episodes, professional liability can be predicated on different bases.

For the Italian penal code, it needs only appear to the health professional from observation and circumstantial data that the minor has been the victim of certain specific crimes provided for by the penal code (sexual violence, physical violence, etc.). The law states that no diagnosis of abuse or neglect is necessary for filing a report, a suspicion is enough. The obligation to report is unconditional and should be performed without undue delay. In these cases, it is a misdemeanor to fail to notify the appropriate authorities. Even when it is an “harmless inaction” (i.e., when, luckily, no further violence episodes occur to the same minor), the health professionals are suitable for criminal punishment. It embraces consideration of the proper scope of the criminal law, and its function in the prevention of harm and the encouragement of socially beneficial conduct and effectiveness of the criminal sanction. Failure to report in certain cases, specified by the Italian penal code, represents a typical inchoate crime and imposes liability where the offender causes no harm.

Furthermore, when resulting harm occurs from the omitted report (i.e., further episodes of violence, personal injury, and death), malpractice claim for negligently failing to diagnose a case of minor may arise. Since maltreatment and abuse may be recognized as medical diagnosis, the failure to recognize them is essentially similar to other cases of malpractice liability for a missed diagnosis.

Within the Italian legal framework, sanctions for manslaughter or personal injury are provided for the Criminal Code. As generally stated by the Italian Criminal Code, it is necessary to demonstrate that a human act produced the personal injuries or the death of the victim. It requires the demonstration of the several inescapable elements: fault, injury or death, and causal link between the fault and the negative events. A specific legislative provision was defined by the law 24/2017 (the so—called “legge Gelli”) (26) in cases when such events are due to professional health care liability, introducing a special, autonomous offense of criminal liability of the medical professionals. The law introduced an exemption from punishment for healthcare professionals when the manslaughter or the personal injury are due to “unskillfulness,” in the case that the existing guidelines or the good clinical practices are adhered to, if these are adequate, in relation to the specificity of the case. No exemption is provided when the events (manslaughter or personal injury) occurred during health care provision were due to negligence and imprudence. Therefore, for the purpose of the special exemption from punishment, as stated by the Gelli law, it must be verified that the fact is due to unskillfulness, that the recommendations provided for by the guidelines (as defined and published by law) or, failing these, the clinical good practices have been complied with, and, finally, that these recommendations are appropriate with regards to the specific case.

In the field of civil law, two different forms of liability are provided in the Italian system, differentiating regarding substantive and procedures: contractual and extracontractual liability (26). The first one arises from the failure to implement an obligation (contract); the extra-contractual liability arises from the breach of a generic duty not to cause unjust damage. In the contract based liability, the claimant (the patient or the legal representative) have merely to demonstrate the damages suffered due to the medical treatment, alleging the relevant breach of the contractual duty; the defendant (health care facility) has the full burden to prove that the performance was correctly carried out and that the negative events were caused by an unforeseeable event, unavoidable in the context of ordinary professional care. In the case of extracontractual liability, the burden of proof lies completely on the claimants (26).

Although the procedural steps are different in civil and criminal proceedings, gathering evidence to establish the existence or non-existence of an offense is pivotal in any case of alleged medical liability. When we are dealing with the hypothesis of failure to diagnose a case of maltreated minor, very difficult issues may arise, such as the difficulties of the diagnosis, high complicated problems of proximate causation, and foreseeability of the event.

First of all, the failure to diagnose the maltreatment may, in itself, present problems of proof since the identification of physical abuse can be difficult (27). It has been outlined that many missed opportunities for diagnosing physical abuse can materialize for professionals whose functions involve regular contacts with children and adolescents, as well as those who may only occasionally come into contact with them in their work (28). Most injuries in children are not the result of abuse or neglect. Minor injuries in children are exceedingly common, and accidental events happen frequently to children not caused by maltreatment (28). Furthermore, histories may be misleading or absent; serious injuries may be missed in the absence of outward signs of trauma, and many different conditions may mimic physical abuse (29, 30). In minor injuries, diagnosis can be very difficult in the absence of a history (31). In this regard, some Authors warned of the alarming risk of flawed theories to explain child physical abuse (32). Apart from the legitimate diagnoses that should be considered in the differential diagnosis requiring carefully obtained history, thorough physical examination, imaging studies, laboratory tests, etc., diagnoses that lack scientific support as explanations of injuries (i.e., vitamin D deficiency and Ehlers-Danlos syndrome) (33) and “fabricated diagnoses, such as “dysphagic choking” and “temporary brittle bone disease” (34, 35) may have a negative impact on clinical practice, as possible source of confusion regarding the diagnosis of abuse and maltreatment. A further hindering element is represented by the fact that minor abuse and maltreatment may involve parents; and it may be possible that the parent informant may also be the one who inflicts violence against the minor. In such situations, it is not unlikely for these parents to underreport or refuse to disclose the victimization incidents as a result of shame, denial, or fear of future legal consequences (36, 37).

To get the diagnosis right is pivotal. As in other fields of malpractice actions, the standard of care of the professional conduct comes at issue. At international level, in 2009 the National Institute for Health and Clinical Excellence (NICE) published guidance aims to raise the awareness of healthcare professionals to the alerting features of child maltreatment, and recently updated them (38); overall in the last decade, guidelines on this issue have increasingly become available. Many Italian Scientific Societies have adopted specific protocols; however, establishing a civil or criminal liability in missed diagnosis of child maltreatment could remain difficult. In fact, article 5 of the recently adopted Italian Law 24/2017, regulating professional liability, established that health professionals should comply, as far as possible and within the bounds of each specific case, with the recommendations included in guidelines drafted by public and private healthcare organizations and institutions, as well as scientific societies and technical-scientific associations, registered on the list compiled and regulated by Ministerial decree and updated every 2 years. In the absence of recommendations, healthcare professionals should follow the good clinical practice (39). However, to date, no guidelines have been published by the Ministry of Health nor good clinical practices on the diagnosis and recognition of child maltreatment. Consequently, difficulties may arise when expert witnesses should define the standard of conduct to which a physician must conform, representing that of a reasonable physician under like circumstances.

Furthermore, proving only that the physician's conduct did not meet the standard of care is not enough; a plaintiff must prove further that the failure to provide standard care caused the subsequent injuries. In other words, despite have established that a physician failed to recognize a case of maltreatment according to the standard of care, is it reasonably foreseeable that his/her failure to properly diagnose the abuse would lead to the eventual injuries? Very often, the assault on the victim is not an isolated, atypical event but part of an alarming scenario of repeated beatings and abuse destined to repeat themselves and to become, increasingly, more and more serious. Very often, severe physical abuse is preceded by relatively minor abusive episodes in children and youths, and that prompt detection of minor injuries might help the identification of those minors who are at risk of more serious injuries. In their study, Sheets et al. (40) found that 27.5% of cases of established physical maltreatment were preceded by escalating and repeated violence episodes. More recently, Pierce et al. reported the alarming figure that of the 14 children with available prior medical records, 9 (64%) had sentinel injuries in the form of prior unexplained bruising (41). Other Authors reported that repeat occurrences of violent acts are seen in 30–50% of patients (42). The link between early abusive injury and later severe injury is widely corroborated (43, 44).

Strategies that could be adopted in order to reduce the risk of medical liability related to a missing diagnosis of child abuse need to be directed toward the implementation of the healthcare personnel education and awareness of the “child-abuse red flags,” particularly for physicians and nurses who works at the emergency department. It is advisable the creation of a “Pink Code team” including nurses, pediatricians, psychologists, neuropsychiatrists, radiologists, gynecologists and forensic physicians, to evaluate suspect cases of child abuse. According to the literature and our experience, as this type of violence is often repeatedly perpetrated, it should be created a wider healthcare network, involving also general practitioner and eventually teachers, to guarantee a major protection, especially in those cases of domestic violence.

## Conclusion

Italian legislation outlines that health professionals are legally obligated to report every suspected case of minor maltreatment and abuse. Ensuring that physicians, both those professionals whose functions involve regular contacts with children and adolescents, as well as those who may only occasionally come into contact with them in their work, understand and are properly trained on their duties and responsibilities to report is pertinent for the protection of the minor and for avoiding further violence episodes. It responds to the imperative that the vulnerable (minors) be protected from abuse, and that the state sanctioned by law the omitted reporting of abuse. Beyond this legal duty, health care professionals must be aware that prompt and careful recognition of minor abuse can dramatically change minor abuse outcomes. Lack of knowledge, lack of understanding of the intricacies surrounding minor abuse, limited education in recognizing the signs and symptoms of abuse, may prevent the identification of a maltreated minor, thus regrettably leading to recurrent abuse. When failure to diagnose and report a suspected incident of minor maltreatment results in further infliction of maltreatment by the abusing subject, professional liability on the part of the physician who negligently omitted the diagnosis could be predicated.

## Data Availability Statement

All datasets generated for this study are included in the article/supplementary material.

## Ethics Statement

The processing of the data reported in this paper is covered by the general authorization to process personal data for scientific research purposes granted by the Italian Data Protection Authority (1 March 2012 as published in Italy's Official Journal No. 72 dated 26 March 2012) since the data do not entail any significant personalized impact on data subject and moreover the case discussed in this paper did not involve invasive procedures.

## Author Contributions

ET conceived this article, reviewed the literature, summarized and analyzed the literature data, and drafted the initial manuscript. She critically reviewed the manuscript for important intellectual content and reviewed and revised the manuscript. CT substantially contributed to the conception and design of this article, and analyzed the casuistry data. ST substantially contributed to the analysis of the casuistry data. MD substantially contributed to the conception and design of this article, supported the literature review, critically reviewed the manuscript for important intellectual content, and reviewed, and revised the manuscript. All authors approved the final manuscript as submitted and agree to be accountable for all aspects of the work.

## Conflict of Interest

The authors declare that the research was conducted in the absence of any commercial or financial relationships that could be construed as a potential conflict of interest.

## References

[1] Moody G Cannings-John R Hood K Kemp A Robling M. Establishing the international prevalence of self –reported child maltreatment: a systematic review by maltreatment type and gender. BMC Public Health. (2018) 18:1164. 10.1186/s12889-018-6044-y 30305071 PMC6180456

[2] Toward a Global Indicator on Unidentified Victims in Child Sexual Exploitation Material, Summary Report, ECPAT, INTERPOL. Lyon (2018).

[3] European report on preventing child maltreatment: Summary. World Health Organization (2013).

[4] Bernacchi E Fabris A Zelano M Multi-country Study on the Drivers of Violence Affecting Children. Italian Report. Istituto degli Innocenti Firenze (2016).

[5] Eads K Breaking silence: underreported child abuse in the healthcare setting. Online J Health Ethics. (2013) 9 10.18785/ojhe.0901.01

[6] Flaherty EG Sege RD Griffith J Price LL Wasserman R Slora E From suspicion of physical child abuse to reporting: primary care clinician decision-making. Pediatrics. (2008) 122:611–9. 10.1542/peds.2007-2311 18676507

[7] Paradise JE Bass J Forman SD Berkowitz J Greenberg DB Mehta K. Minimum criteria for reporting child abuse from health care settings. Del Med J. (1997) 69:357–63. 9260386

[8] Newton AS Zou B Hamm MP Curran J Gupta S Dumonceaux C. Improving child protection in the emergency department: a systematic review of professional interventions for health care providers. Acad Emerg Med. (2010) 17:117–25. 10.1111/j.1553-2712.2009.00640.x 20370740 PMC3023813

[9] Gender-related Health in Tuscany Agenzia regionale di sanità della Toscana. (2015) Available online at: https://www.ars.toscana.it/files/pubblicazioni/Rapporti_relazioni_sintesi/salute_genere/Gender-related_Health_in_Tuscany_web3.pdf

[10] Ferrara P Corsello G Basile MC Nigri L Campanozzi A Ehrich. The economic burden of child maltreatment in high income countries. J Pediatr. (2015) 167:1457–9. 10.1016/j.jpeds.2015.09.044 26611458

[11] Ferrara P Gatto A Manganelli NP Ianniello F Amodeo ME Amato M. The impact of an educational program on recognition, treatment and report of child abuse. Ital J Pediatr. (2017) 43:72. 10.1186/s13052-017-0389-1 28806991 PMC5557001

[12] Sege RD Flaherty EG. Forty years later: inconsistencies in reporting of child abuse. Arch Dis Child. (2008) 93:822–4. 10.1136/adc.2006.100545 18539683

[13] Lynne EG Gifford EJ. Barriers to Reporting Child Maltreatment. Do emergency medical services professionals fully understand their role as mandatory reporters? North Carolina Med J. (2015) 76:13–8. 10.18043/ncm.76.1.13 25621471

[14] Anderst J Nielsen-Parker M Moffatt M Frazier T Kennedy C. Using simulation to identify sources of medical diagnostic error in child physical abuse. Child Abuse Negl. (2016) 52:62–9. 10.1016/j.chiabu.2015.12.015 26779947

[15] Sitting JS Uiterwaal CS Moons KG Russel IM Nievelstein RA Nieuwenhuis EE. Value of systematic detection of physical child abuse at emergency rooms: a cross-sectional diagnostic accuracy study. BMJ Open. (2016) 22:e010788. 10.1136/bmjopen-2015-010788 27006346 PMC4809108

[16] Anderst J Kellogg N Jung I. Is the diagnosis of physical abuse changed when child protective services consults a child abuse pediatrics subspecialty group as a second opinion? Child Abuse Neglect. (2009) 33:481–9. 10.1016/j.chiabu.2009.05.001 19766309

[17] McGuire L Martin KD Leventhal JM. Child abuse consultations initiated by child protective services: the role of expert opinions. Acad Pediatrics. (2011) 11:467–73. 10.1016/j.acap.2011.06.005 21820376

[18] Jones R Flaherty EG Binns HJ Price LL Slora E Abney D Clinicians'description of factors influencing their reporting of suspected child abuse: report of the child abuse reporting experience study research group. Pediatrics. (2008) 122:259–66. 10.1542/peds.2007-2312 18676541

[19] Talsma M Bengtsson Boström K Östberg AL. Facing suspected child abuse – what keeps swedish general practitioners from reporting to child protective services? Scand J Prim Health Care. (2015) 33:21–6. 10.3109/02813432.2015.1001941 25676563 PMC4377737

[20] Romeo L Gibelli D Giannotta F Zocchi MT Rossi RC Kustermann A. Can family pediatricians in italy identify child abuse? A survey. Minerva Pediatr. (2016) 68:230–6. 27176667

[21] Louwers EC Korfage IJ Affourtit MJ Scheewe DJ van de Merwe MH Vooijs-Moulaert AF. Effects of systematic screening and detection of child abuse in emergency departments. Pediatrics. (2012) 130:457–64. 10.1542/peds.2011-3527 22926179

[22] Benger JR Pearce A. Simple intervention to improve detection of child abuse in emergency departments. BMJ. (2002) 324:780–2. 10.1136/bmj.324.7340.780 11924662 PMC1122710

[23] Levi BH Portwood SG. Reasonable suspicion of child abuse: finding a common language. J Law Med Ethics. (2011) 39:62–9. 10.1111/j.1748-720X.2011.00550.x 21314795

[24] Levi BH Crowell K. Child abuse experts disagree about the threshold for mandated reporting. Clin Pediatr (Phila). (2011) 50:321–9. 10.1177/0009922810389170 21138854

[25] McTavish JR Kimber M Devries K Colombini M MacGregor JCD Wathen CN. Mandated reporters' experiences with reporting child maltreatment: a meta-synthesis of qualitative studies. BMJ Open. (2017) 7:e013942. 10.1136/bmjopen-2016-013942 29042370 PMC5652515

[26] Zerbo S Malta G Argo A. Guidelines and current assessment of health care responsibility in Italy. Risk Manag Healthcare Policy. (2020) 13:183–9. 10.2147/RMHP.S238353 32210649 PMC7073368

[27] Jacobi G Dettmeyer R Banaschak S Brosig B Herrmann B. Child abuse and neglect: diagnosis and management. Dtsch Arztebl Int. (2010) 107:231–40. 10.3238/arztebl.2010.0231 20396522 PMC2855177

[28] Christian CW Committee on Child Abuse and Neglect, American Academy of Pediatrics. The evaluation of suspected child physical abuse. Pediatrics. (2015) 135:e1337–54. 10.1542/peds.2015-0356 25917988

[29] Pomeranz ES. Child abuse and conditions that mimic it. Pediatr Clin North Am. (2018) 65:1135–1150. 10.1016/j.pcl.2018.07.009 30446053

[30] Christian CW States LJ. Medical mimics of child abuse. AJR Am J Roentgenol. (2017) 208:982–90. 10.2214/AJR.16.17450 28225649

[31] Jenny C Hymel KP Ritzen A Reinert SE Hay TC. Analysis of missed cases of abusive head trauma. JAMA. (1999) 281:621–6. 10029123 10.1001/jama.281.7.621

[32] Leventhal JM Edwards GA. Flawed theories to explain child physical abuse. What are the medical-legal consequences? JAMA. (2017) 318:14. 10.1001/jama.2017.11703 28975235

[33] Castori M. Ehlers-Danlos syndrome(s) mimicking child abuse: is there an impact on clinical practice? Am J Med Genet C Semin Med Genet. (2015) 169:289–92. 10.1002/ajmg.c.31460 26452443

[34] Edwards GA. Mimics of child abuse: can choking explain abusive head trauma? J Forensic Leg Med. (2015) 35:33–37. 10.1016/j.jflm.2015.06.012 26344456

[35] Marcovitch H Mughal MZ. Cases do not support temporary brittle bone disease. ActaPaediatr. (2010) 99:485–6. 10.1111/j.1651-2227.2010.01693.x 20096023

[36] Chan KL. Are parents reliable in reporting child victimization? Comparison of parental and adolescent reports in a matched chinese household sample. Child Abuse Negl. (2015) 44:170–83. 10.1016/j.chiabu.2014.11.001 25465317

[37] Compier-de Block LHCG Alink LRA Linting M van den Berg LJM Elzinga BM Voorthuis A. Parent-child agreement on parent-to-child maltreatment. J Fam Violence. (2017) 32:207–17. 10.1007/s10896-016-9902-3 28163367 PMC5250653

[38] NICE guidelines Child maltreatment: when to suspect maltreatment in under 18s. Clinical guideline. Last updated October 2017 (2009). Available online at: www.nice.org.uk/guidance/cg

[39] Albolino S Bellandi T Cappelletti S Di Paolo M Fineschi V Frati P. New rules on patient's safety and professional liability for the italian health service. Curr Pharm Biotechnol. (2019) 20:615–24. 10.2174/1389201020666190408094016 30961486

[40] Sheets LK Leach ME Koszewski IJ Lessmeier AJ Nugent M Simpson P. Sentinel injuries in infants evaluated for child physical abuse. Pediatrics. (2013) 131:701–7. 10.1542/peds.2012-2780 23478861

[41] Pierce MC Kaczor K Acker D Webb T Brenzel A Lorenz DJ History, injury, and psychosocial risk factor commonalities among cases of fatal and near-fatal physical child abuse. Child Abuse Negl. (2017) 201:263–77. 10.1016/j.chiabu.2017.04.033 28500923

[42] Kocher MS Kasser JR. Orthopaedic aspects of child abuse. J Am Acad Orthop Surg. (2000) 8:10–20. 10.5435/00124635-200001000-00002 10666649

[43] Putnam-Hornstein E Cleves MA Licht R Needell B. Risk of fatal injury in young children following abuse allegations: evidence from a prospective, population-based study. Am J Public Health. (2013) 103:e39–e44. 10.2105/AJPH.2013.301516 23947328 PMC3780759

[44] Petska HW Sheets LK. Sentinel injuries: subtle findings of physical abuse. Pediatr Clin North Am. (2014) 61:923–35. 10.1016/j.pcl.2014.06.007 25242706

